# Biodegradable and injectable curcumin-loaded hydrogel for the prevention of postoperative intrauterine adhesion

**DOI:** 10.3389/fbioe.2025.1640440

**Published:** 2025-11-17

**Authors:** Qingmei Yang, Han Xu, Yang Chen, Xiaofen Jin, Qing Wu

**Affiliations:** 1 Department of Gynecology, Yibin First People’s Hospital, Yibin, China; 2 Center for Reproductive Medicine, Department of Gynecology, Zhejiang Provincial People’s Hospital (Affiliated People’s Hospital, Hangzhou Medical College), Hangzhou, Zhejiang, China; 3 Zhejiang Key Laboratory of Precision Diagnosis and Therapy for Major Gynecological Diseases, Women’s Hospital, Zhejiang University School of Medicine, Hangzhou, Zhejiang, China

**Keywords:** intrauterine adhesion, curcumin, biodegradable, injectable, hydrogel

## Abstract

Intrauterine adhesions (IUAs) are essentially fibrosis of the endometrium within the uterine cavity. It is a common cause of infertility in women and seriously affects their physical and mental health. Current therapeutic strategies have failed to reach satisfactory outcomes. Injectable and self-healing uterine hydrogels with antifibrotic properties would be efficient in preventing IUA. In this work, we prepared curcumin-loaded carboxymethyl chitosan (CMC)–oxidized hyaluronic acid (OHA) intrauterine hydrogel (Cur@CMC-OHA hydrogel) with antifibrotic properties, and its injectable and self-healing properties could be adapted to the morphostructures of the uterine cavity. The hydrogels exhibited tissue adhesive power, which is ideal for stable uterine cavity retention and therapeutic outcomes. *In vivo* experiments showed that *in situ* injection of the Cur@CMC-OHA hydrogel into a mouse model of IUA reduced fibrotic tissues, prevented IUA, and improved the reproductive outcomes. It effectively downregulated fibrosis-associated transforming growth factor-β1 (TGF-β1) expression and reversed epithelial–mesenchymal transition (EMT), resulting in anti-fibrotic and fertility restoration. In conclusion, Cur@CMC-OHA hydrogel may be a promising alternative for clinical treatment of uterine adhesion.

## 1 Introduction

Intrauterine adhesions (IUAs) are characterized by the deposition of fibrotic tissues in the uterine cavity as a result of endometrial damage caused mainly by surgical interventions such as curettage and hysteroscopy (Yu et al., 2008; Hooker et al., 2013). The development of IUA is associated with complications of insufficient menstruation, amenorrhea, cyclic pain, and even infertility (Di Spiezio Sardo et al., 2016). In women of reproductive age, 15%–20% of patients undergoing curettage develop IUA, which are associated with infertility due to severe damage to the endometrial basal layer (Gilman et al., 2016; Dreisler and Kjer, 2019). Although standardized trans-hysteroscopic resection of uterine adhesions (TCRA) has been clinically effective in the management of IUA, pregnancy rates are still far from satisfactory due to severe clinical conditions of readhesion. The rate of IUA recurrence after surgical separation of severe adhesions has been reported to be as high as 62.5% (Bosteels et al., 2017). A number of methods have been proposed to prevent the recurrence of IUA, including placement of intrauterine devices (IUDs) and estrogen therapy (Lin et al., 2013). However, these methods have shown limited clinical efficacy, and reconstruction of the functional endometrium in moderate and severe cases remains a major challenge. Therefore, there is an urgent need for development of a safe and efficient anti-adhesion material to effectively prevent the recurrence of IUA.

Injectable hydrogels are a promising strategy for the prevention of IUA recurrence because such hydrogels can be constructed *in situ* by various physical, chemical, and ion-mediated gelation methods (Liu et al., 2018; Wang et al., 2020; Li et al., 2024). Injectable hydrogels exhibit liquid-like properties in some cases, leaving them in the gel state and uterine cavities completely filled. Based on the special morphology and physiological structure of the uterine cavity, the intrauterine hydrogel should be adapted to the uterine cavity morphology and have a certain degree of tension resistance. The necessity of the injectable property ensures that the gel fills the entire uterine cavity. Advanced injectable cream-like hydrogels with multiple functionalities, including rapid gelation, self-healing, antioxidation, anti-inflammation, and anti-cell adhesion, have been developed to prevent postoperative abdominal adhesions (Liu et al., 2023). Injectable hydrogels with multiple functionalities such as anti-endometrial fibrosis, physical barriers, antioxidant capacity, and self-healing should be a promising method to prevent postoperative intrauterine adhesion.

Curcumin (Cur) represents the major curcuminoid analog of turmeric rhizomes (*Curcuma longa* L., family Zingiberaceae), which is responsible for the dark yellow color of turmeric (Akbik et al., 2014; Amalraj et al., 2017). Since then, the pharmacological effects of curcumin have been extensively studied. The antibacterial properties of curcumin were reported in 1949; other studies focused on the multiple actions of curcumin, such as anti-infective, anti-inflammatory (Edwards et al., 2017), antioxidant (SCHRAUFSTATTER and BERNT, 1949), anticarcinogen (Weng and Goel, 2022; Kuttan et al., 1985), immunomodulatory, and wound healing effects (Gong et al., 2023). In recent years, numerous studies have gradually recognized the unique advantages of the antifibrotic effects exerted by curcumin on the lungs and liver. However, curcumin’s low stability, insufficient water solubility, rapid decomposition, and poor bioavailability greatly limit its current clinical application. Endometrial fibrosis is an important factor in the development of IUA, and it remains to confirm whether Cur has an antifibrotic effect (Ma et al., 2019). Oxidized hyaluronic acid (OHA) is the product of the oxidation of hyaluronic acid, and the active aldehyde group in its molecule can self-assemble with the amino group in carboxymethyl chitosan (CMC) in a Schiff base reaction to form a hydrogel. This self-assembled uterine hydrogel exhibits good injectability, self-healing ability, biodegradability, biocompatibility, certain compressive strength, and adhesiveness and gradually breaks the Schiff base bond, thereby releasing the drug slowly.

**SCHEME 1:**
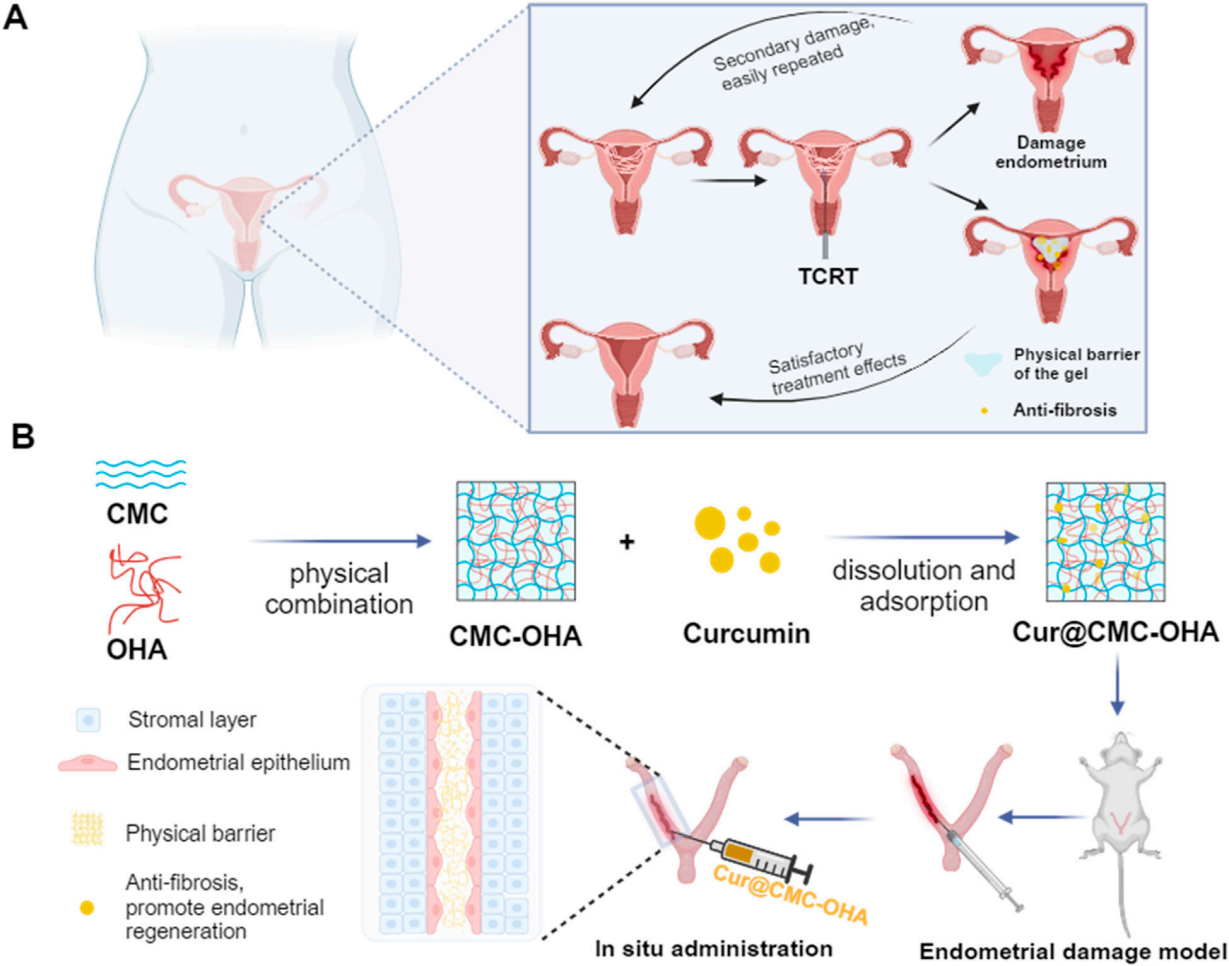
Design of an injectable and self-healing hydrogel with antifibrotic properties for preventing IUA. The hydrogel is prepared using CMC and OHA. The curcumin-loaded hydrogel is injected into the uterine cavity, enabling the slow release of the drug and downregulation of TGF-β1 expression, thereby modulating extracellular matrix (ECM) deposition and myofibroblast activation.

The aim of the present study was to prepare an injectable and self-healing curcumin-loaded hydrogel through a Schiff base reaction between CMC and OHA (Cur@CMC-OHA hydrogel) to prevent uterine adhesion and anti-endometrial fibrosis, enabling continuous release of curcumin to downregulate TGF-β1 expression, thereby modulating extracellular matrix (ECM) deposition and myofibroblast activation to prevent IUA (Scheme 1A,B). In this work, unlike the anti-adhesion membranes used now, the hydrogel can be immediately fixed on any irregular surface due to its injectability and malleability, facilitating local application. It also has the ability to resist compression of uterine contractions and adheres well to tissue surfaces with good biocompatibility and biodegradability. The efficacy of the Cur@CMC-OHA hydrogel for preventing IUA was evaluated in a mouse model of IUA, where it prevented uterine fibrosis by promoting reepithelialization of the damaged endometrium and increasing pregnancy rates.

## 2 Materials and methods

### 2.1 Materials, cell lines, and animals

Oxidized hyaluronic acid (molecular weight 100–200 KD and oxidation rate 48.2%) and carboxymethyl chitosan (molecular weight 10–20 W, BR, and water solubility 60%–100%) were purchased from Sigma-Aldrich (Shanghai, China). All other chemical reagents were of analytical grade and were not further purified. Ishikawa cells (IK cells) were human endometrial adenocarcinoma cells purchased from the National Cell Line Resource Centre (Beijing, China). IK cells were separately cultured in Dulbecco’s modified Eagle’s medium (DMEM, Gibco, United States), containing 10% fetal bovine serum (FBS, Gibco, United States) (DMEM, Gibco, United States) at 37 °C and 5% humidity. Animal health and use procedures were approved by the Animal Husbandry and Zhejiang Sci-Tech University Ethics Committee (Approval No. ZH20231029033) and complied with all relevant agency and government regulations to ensure the ethical use of animals. Six-week-old ICR female/male mice (30 ± 2 g) were purchased from Hangzhou Hangsi Biotechnology Co., LTD. and acclimatized to the new environment for at least 1 week. The mice were raised in SPF conditions. Female mice underwent vaginal smear tests at 08:00 every morning to assess the estrus cycle. Animal models were established using mice with 4 consecutive days of the estrus cycle. Mechanical damage of the uterine cavity was induced to simulate IUA during the late estrus period. Mice were anesthetized by intraperitoneal injection of tribromoethanol. An incision was made below the midline of the abdomen to expose the uterus. A 7-gauge needle was inserted into the left and right uterine joints, and the endometrium was carefully scraped back and forth until the uterus is visibly congested. Then, the abdominal cavity was closed under sterile conditions. The control group did not receive surgery. The treatment group received intrauterine injection of 50 μL Cur@CMC-OHA. The IUA group received 50 μL PBS. Each group consisted of 12 mice. The uterus of each mouse was collected for subsequent experiments.

### 2.2 Preparation and characterization of the Cur@CMC-OHA hydrogel

CMC-OHA hydrogels were prepared by cross-linking CMC with OHA. In brief, CMC and OHA were dissolved in double-distilled water so that the mass concentrations of CMC were 5, 10, or 15 mg/mL and the mass concentrations of OHA were 1, 2, or 4 mg/mL and then stirred overnight. A cross-linked blank hydrogel was prepared by mixing the CMC and OHA solutions in a 1:1 volume ratio. Curcumin-loaded CMC-OHA hydrogels (Cur@CMC-OHA hydrogels) were prepared by dissolving curcumin directly in the previously prepared CMC solution, stirring with a magnetic stirrer overnight, and then mixing Cur@CMC and OHA solutions in a 1:1 volume ratio. The gelation time was measured using the tilt method. After mixing the CMC and OHA solutions, a timer was started, and the vial was tilted every 5 s to observe the mixed solution until the liquid stopped flowing; the gelation time was then recorded. The characteristic infrared peaks of Cur, CMC, OHA, CMC-OHA, and Cur@CMC-OHA were determined.

### 2.3 Morphology

The morphological characteristics of CMC-OHA and Cur@CMC-OHA hydrogel were characterized by scanning electron microscopy (SEM). The hydrogels were allowed to crosslink and form hydrogels at room temperature. The hydrogels were then freeze-dried and freeze-fractured in liquid nitrogen. A thin layer of gold was applied to the surface of the cross-section before observation. The surface and cross-section morphology were observed using a scanning electron microscope (JSM-5900LV, JEOL, Japan).

### 2.4 *In vitro* swelling rate and degradation time assays

To test the swelling rate of the hydrogels, 500 µL of hydrogels was immersed in 5 mL of PBS solution and incubated at 37 °C. After incubation for 0.5, 1, 2, 4, 6, 8, 10, 12, and 24 h, the hydrogels were removed and gently dried on a filter paper. The weight was measured, and the swelling rate was calculated using the formula: (where W_0_ is the initial weight of each sample and W_t_ is the weight after different times of incubation). To test the degradation curve, 500 μL of the hydrogel was immersed in 5 mL of PBS solution and incubated at 37 °C. After equilibrium swelling, the incubation was continued for 1, 2, 3, 4, 5, 6, 7, 8, 9, 10, 12, and 14 days, and the hydrogels were removed at the same time each day and gently dried on a filter paper. The weight was measured, and the degradation curve was recorded (n = 3).

### 2.5 Rheological analysis

Rheological property analysis of CMC-OHA hydrogels was carried out using a HAAKE MARS RS6000 rheometer (Thermo Scientific, Germany) in the oscillatory mode at 37 °C. The rheology of the CMC-OHA hydrogels was measured using a double syringe equipped with a mixing chamber to mix 300 mL of CMC and OHA. In brief, 300 mL each of CMC and OHA were mixed using a double syringe equipped with a mixing chamber. The sample was then placed on the rheometer, and the upper plate was lowered to a gap size of 1 mm. The storage modulus (G′) and loss modulus (G″) were recorded as a function of time. The gelation time is the time at which G′ is higher than G″.

### 2.6 Adhesion and form-fitting performance tests

The adhesion performance of the hydrogels to different materials was characterized as follows: the organic material pigskin and the inorganic material rubber were chosen as the substrates for visual inspection. Adhesion and form-fitting performance tests were conducted as follows: first, fresh pig skin was used as the substrate, which was soaked in alcohol to remove the fat layer, and then cut into rectangles of 35 mm × 30 mm and soaked in PBS for use. Hydrogel was applied on the surface of the broken ends of the pig skin. The blocks of breeder pig skin were then glued, stretched, and glued again. Then, an appropriate amount of the prepared hydrogel was applied to the joint of the index finger, and the index finger was flexed to 0° and 90° to observe the breaking and sliding of the hydrogel.

The uterus was exposed by making a 1.5-cm incision in their lower abdomen. The hydrogel was injected into the uterus with a syringe through a catheter (1 mm) until it fills the uterus. After 10 min, the mouse uterus was opened, and the hydrogel was removed to observe the morphology.

### 2.7 Injectability and self-healing performance testing

To prepare CMC-OHA hydrogels stained with different dyes, a pre-gel solution mixed with Reichun red dye was drawn up with a 1-mL syringe, and after the solution was gelatinized, the hydrogel was squeezed out by pressing the syringe and deposited onto paper.

The hydrogel was injected into molds to form a blueberry-shaped and star-shaped hydrogel, and then the two differently colored hydrogels were crushed, put into the star-shaped mold again, placed in a humid environment to repair again for 30 min, and then removed for observation. Two pieces of hydrogels of different colors were cut into two parts with a scalpel, cross-fitted together, put into a Petri dish containing saline for self-healing, and then removed for a period of time to observe the healing condition.

### 2.8 *In vitro* cytotoxicity assay

The cytotoxicity of Cur@CMC-OHA and CMC-OHA was assessed using IK cells and CCK-8. The hydrogels were sterilized under UV light for 30 min prior to testing. The hydrogels Cur@CMC-OHA and CMC-OHA (200 mg) were then placed in fresh cell culture medium (10 mL) overnight (12 h) to obtain the leachate (1X). Cur@CMC-OHA and CMC-OHA (1 g) were placed into fresh medium (10 mL) until complete degradation to obtain the degradation solution. It was then diluted to different concentrations (20 mg/mL, 2 mg/mL, 0.2 mg/mL, and 0.02 mg/mL). IK cells were inoculated into 96-well plates (5,000 cells per well) and cultured at 37 °C and 5% CO_2_. After cell adhesion, the cell culture medium was replaced with different concentrations of leachate and degradate. After 24 and 48 h, 10% CCK-8 was added to each well. The absorbance of the sample solution was measured at 450 nm using an enzyme marker (SuperMax 3100, Shanghai Shanpu Biotechnology Co., Ltd.). Metabolic activity of more than 70% was considered non-toxic.

### 2.9 *In vivo* toxicity assessment

To assess the possible side effects of Cur@CMC-OHA hydrogel treatment in female mice, all mice were observed for general conditions (activity, energy, hair, feces, behavioral patterns, and other clinical signs), body weight, and mortality after administration of the Cur@CMC-OHA hydrogel. At the end of the treatment, major organs (heart, liver, spleen, lungs, kidneys, and brain) were removed and immediately fixed in 4% paraformaldehyde. These tissues were examined histopathologically using hematoxylin and eosin (HE) staining.

### 2.10 Histological analysis

After 7 and 14 days of surgery, mice were executed, and tissues were embedded in standard paraffin, sectioned, and stained with HE. Morphological changes were observed under a light microscope. Five fields of view were selected for each image for counting. Image Pro-Plus 6.0 (IPP 6.0) was applied to analyze endometrial thickness, total number of endometrial glands, and the area of endometrial interstitial fibrosis. Endometrial fibrosis was observed using Masson’s trichrome staining. In brief, mice were executed 10 days after surgery, tissues were embedded in standard paraffin, and 4-μm serial sections were prepared. Sections were immunolabeled with the anti-TGF-β1 antibody (1:150, rabbit, Bioss, Beijing, China) and anti-α-SMA antibody (1:50, mouse, Boster, Wuhan, China). The percentage of positively stained areas was quantified using Image-Pro Plus software (Media Cybernetics, Rockville, MD).

### 2.11 Western blot

Proteins were extracted from mouse uterine tissue by adding phosphatase and protease inhibitors with RIPA lysis buffer (Bain-marie, China). The extraction process was performed on ice. Proteins were then detected using the BCA protein quantification kit. The tissue lysate was then diluted at a ratio of 1:5 with protein sampling buffer (5×) and heated at 100 °C for 5 min. Protein extracts were separated using a sodium dodecyl sulfate–polyacrylamide gel electrophoresis (SDS-PAGE, 4%–20%) gradient and then transferred onto polyvinylidene difluoride (PVDF) membranes (Merck Millipore, Germany) blocked with 5% skimmed milk for 2 h. The membranes were incubated overnight at 4 °C with primary antibodies for E-cadherin (Lot No. 1038733-7), N-cadherin (Lot No. 0049320969) (rabbit monoclonal antibody, 1:100; Abcam, Cambridge, UK), TGF-β1 (Lot No. 0051090201), Smad3 (Lot No. 3522070638), vimentin (Lot No. 400000086) (rabbit monoclonal antibody, 1:100; ABclonal, Wuhan, China), and alpha-smooth muscle actin (α-SMA) (Lot No. 4000000298, rabbit monoclonal antibody, 1:200; ABclonal, Wuhan, China), followed by 2 h of incubation with secondary antibodies. Finally, specific protein bands were detected using a chemiluminescence and gel imaging system (Bio-Rad, United States). The intensity of the bands was quantified using ImageJ software.

### 2.12 Statistical analysis

Statistical calculations were performed using SPSS 26. Data are presented as mean ± standard deviation (SD). Multiple comparisons were statistically analyzed using one-way analysis of variance (ANOVA), followed by Tukey’s and Dunnett’s *post hoc* tests. Statistical analyses between two groups of data were performed using Student’s t-test. Values of p < 0.05 were considered statistically significant differences (*P < 0.05, **P < 0.01, ***P < 0.001, and ****P < 0.0001).

## 3 Results and discussions

### 3.1 Preparation and characterization of Cur@CMC-OHA hydrogels

The proposed curcumin-loaded hydrogels were prepared using CMC and OHA. The aldehyde group of oxidized hyaluronic acid can react with the amino group of carboxymethyl chitosan as a polysaccharide cross-linking agent to form Schiff base bonds and hydrogels, Moreover, ionic bonding exists between the negative charge of hyaluronic acid and the positive charge of carboxymethyl chitosan as positive and negative charges are attracted to each other (Zhong et al., 2019).

**FIGURE 1:**
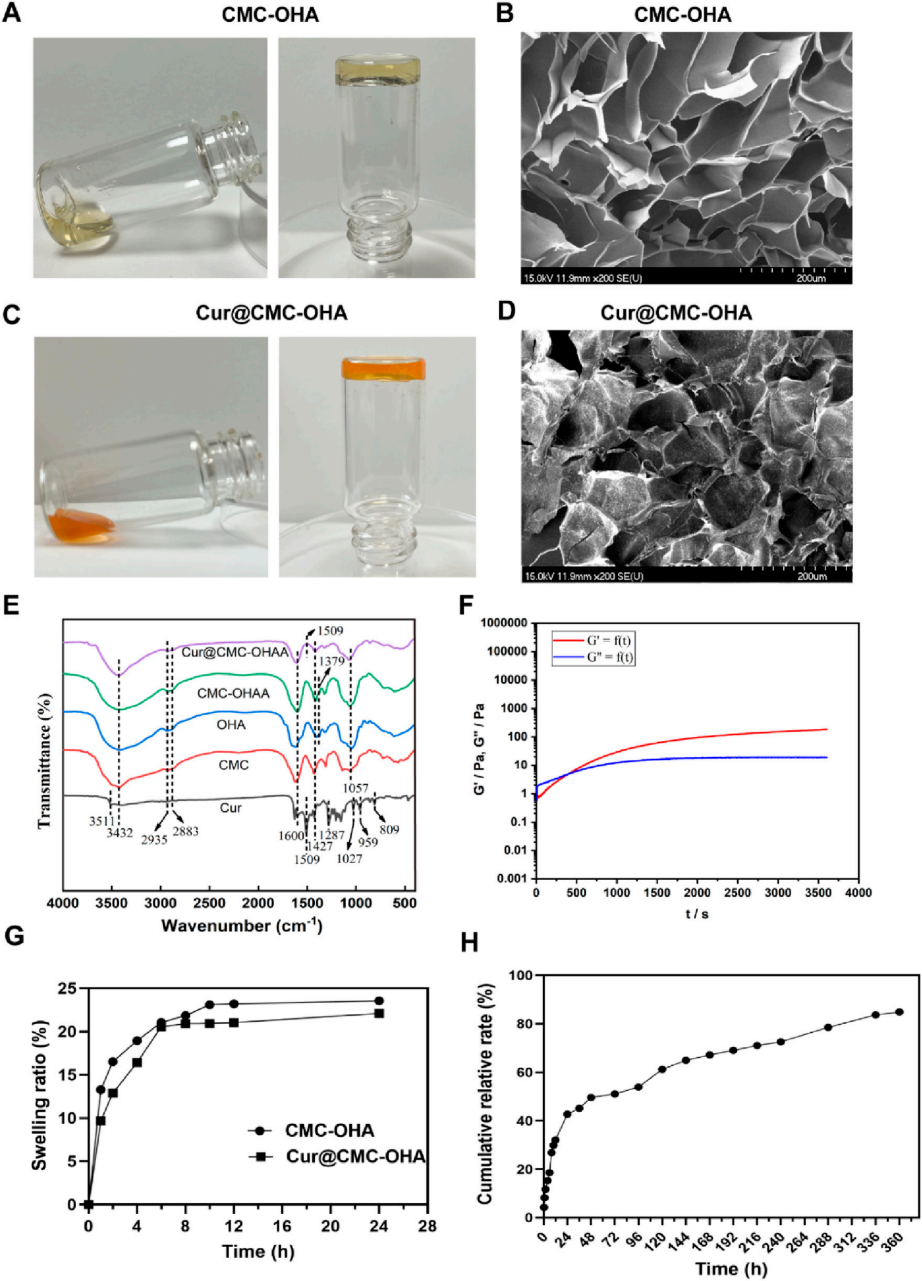
**(A)** Appearance of CMC-OHA hydrogel; **(B)** microstructure of CMC-OHA hydrogel; **(C)** appearance of Cur@CMC-OHA hydrogel; **(D)** microstructure of Cur@CMC-OHA hydrogel; **(E)** FTIR spectra of Cur, CMC, OHA, CMC-OHA hydrogel, and Cur@CMC-OHA hydrogel; **(F)** rheological characteristics of Cur@CMC-OHA hydrogel; **(G)** *in vitro* dissolution study of CMC-OHA/Cur@CMC-OHA hydrogels in PBS solution at pH 7.4; **(H)** cumulative release profile of Cur in Cur-loaded CMC-OHA hydrogels in the presence of 10 units/mL hyaluronidase (n = 3).

**FIGURE 2:**
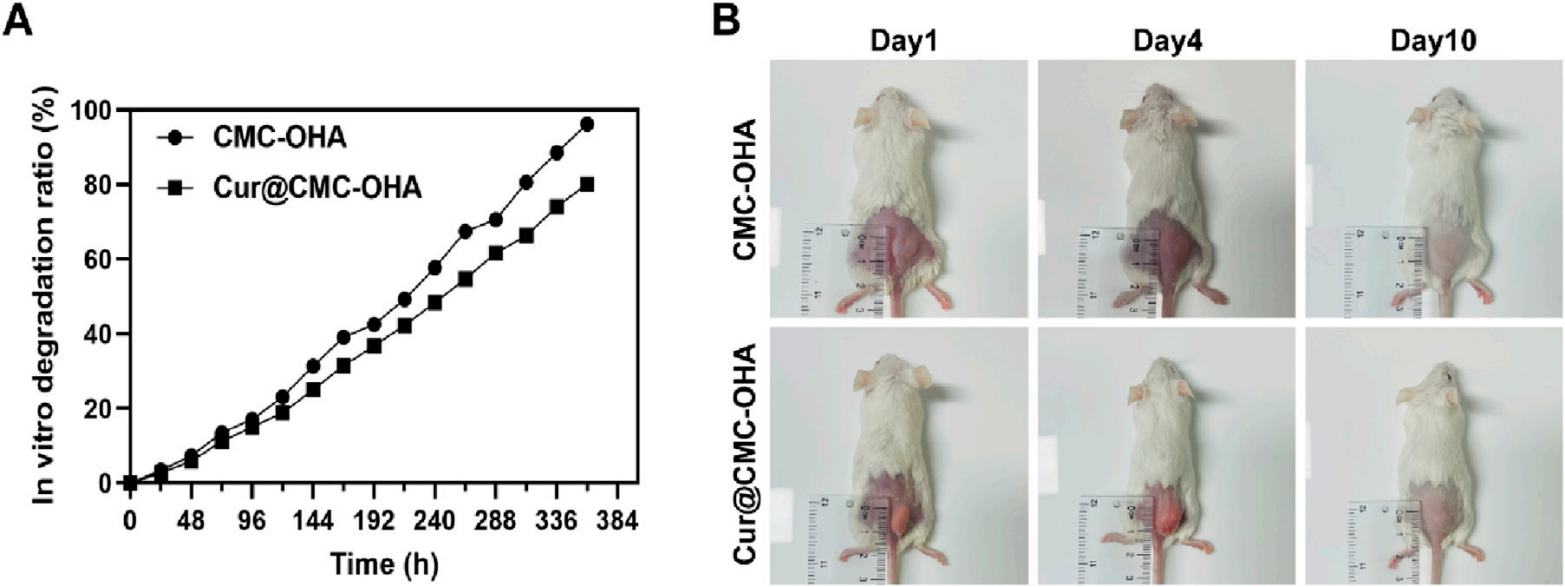
**(A)** *In vitro* degradation kinetics of CMC-OHA/Cur@CMC-OHA hydrogel in 10 units/mL hyaluronidase solution at 37 °C. **(B)** *In vivo* subcutaneous degradation of hydrogels in ICR mice (n = 3).

To find the optimal ratio for the subsequent experiments, different concentrations of CMC and OHA were mixed in equal volumes to make a series of hydrogels. Supplementary Table S1 shows that after mixing different concentrations of CMC and OHA, C10O4, with a gel formation time of 350 s, was used for the subsequent experiments due to the difference in the length of the gelation time, taking into account the operator’s handling time required after the uterine operation. The hydrogel structure remained unchanged after loading curcumin (Figure 1A,C). The chemical structures of OHA and CMC-OHA hydrogel were characterized using FTIR. As shown in Figure 1E, the CMC-OHA hydrogel showed characteristic peaks of OHA near 1,379 cm^-1^, and Cur@CMC-OHA hydrogel showed characteristic peaks of Cur and CMC near 1,509 cm^-1^, 1,600 cm^-1^, and 1,427 cm^-1^, indicating successful synthesis of the material. The rheological properties of the hydrogels were characterized using a rheometer. The storage modulus (G’) and loss modulus (G’) of the prepared hydrogels were studied over time (Figure 1F). Meanwhile, the gel–sol transition time (350 s) was determined at the intersection of G′ and G″. SEM analyses showed that the CMC-OHA and Cur@CMC-OHA hydrogels exhibited a porous three-dimensional network structure inside the hydrogel, with all the pores connected to each other, and the pore sizes ranging from 60 to 100 μm; the curcumin drug was uniformly distributed in the pores (Figures 1B,D). The porous structure facilitates fluid absorption and retention in humid environments while allowing nutrient exchange and metabolic waste transfer (Lv et al., 2021; Yao et al., 2019). In addition, the interconnected cavities provide support for the hydrogel to maintain a certain morphology. After intrauterine application of hydrogels, they absorbed body fluids, thereby increasing their volume, ensuring that all injured tissues can be effectively covered by the hydrogel (Cai et al., 2019). Therefore, hydrogel swelling is desirable in the prevention of IUA. As shown in Figure 1D, Cur@CMC-OHA hydrogel, a hydrogel loaded with curcumin, had an unchanged hydrogel structure and possessed good solubility, and the drug was uniformly adhered to the surface of the hydrogel and between the voids. As the hydrogel degraded, the drug was slowly released. Figure 1G shows that CMC-OHA and Cur@CMC-OHA hydrogels exhibited solubility (20.18% ± 3.44% for CMC-OHA and 18.06% ± 4.29% for Cur@CMC-OHA), suggesting that they could cover the damaged tissue without disrupting the normal morphology of the uterus due to excessive solubility. Figure 1H shows the cumulative release profile of Cur in Cur-loaded CMC-OHA hydrogels in the presence of 10 units/mL hyaluronidase; the cumulative release test showed that the hydrogel could release the drug slowly *in vitro*, and more than 80% of the drug could be released in 15 days. Second, degradability is another important property; therefore, to verify the degradability of the hydrogels, Cur@CMC-OHA hydrogels were immersed in PBS containing hyaluronidase, placed on a 37° shaker (70rpm), and the weight changes were recorded. Figure 2A shows that all the formulations underwent degradation after 14–20 days, and the drug release was up to 80% at 15 days. Figure 2B shows that *in vivo* degradation experiments were also carried out subcutaneously on the back of mice with the same trend. This effectively eliminates the need for a second surgery to remove the nondegradable material. On day 4, the size of the gel in the body was larger than that on day 1, likely due to its penetration into the surrounding tissue, and it was finally degraded without causing tissue damage after 10 days.

### 3.2 Injectability, shape adaptation, and adhesion properties of hydrogels

**FIGURE 3:**
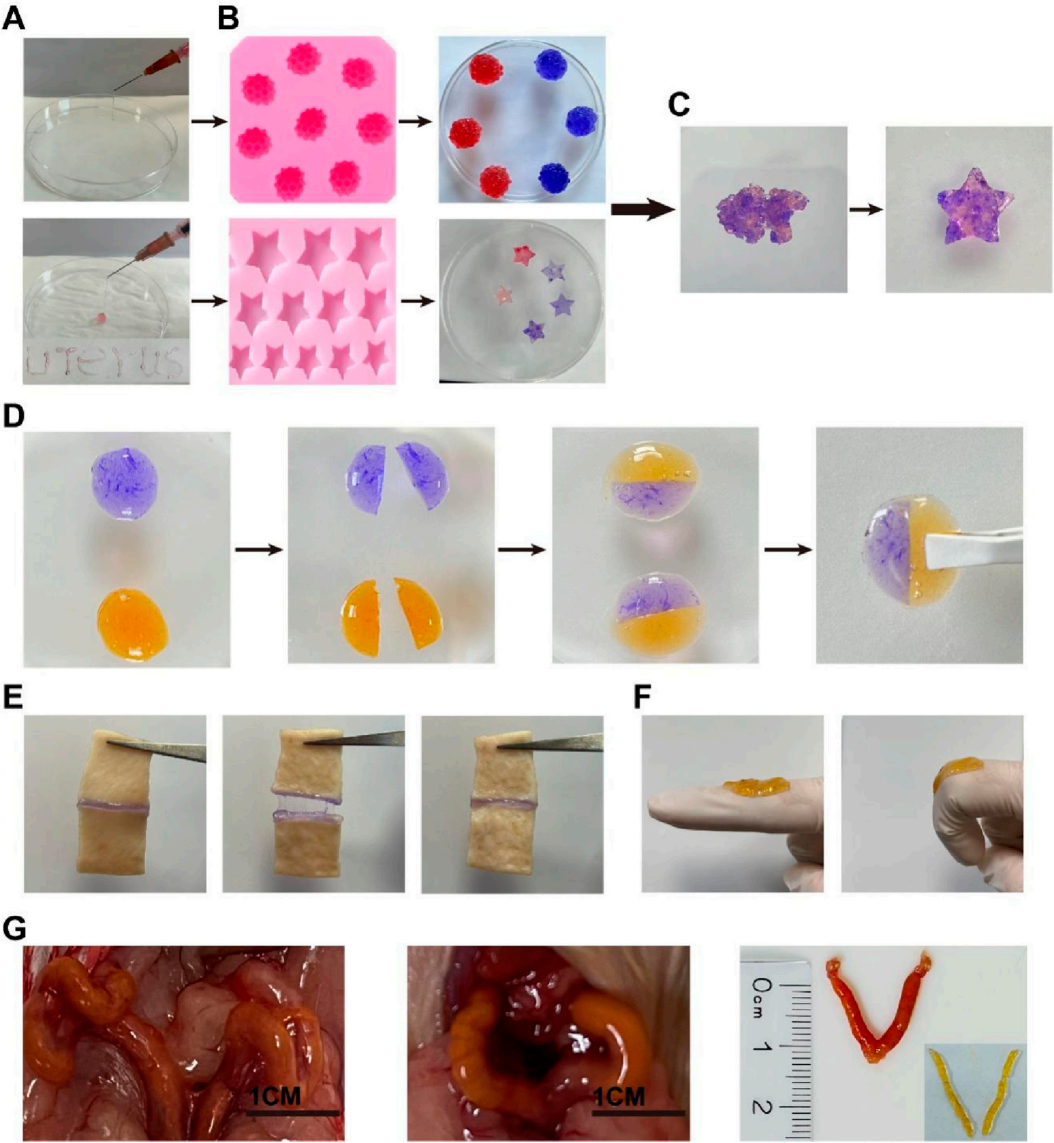
**(A)** Hydrogel injected with a syringe and deposited onto paper; **(B)** plasticity of the hydrogel after injection into different molds; **(C)** self-healing hydrogel after being cut into pieces and placed in a starfish mold for 30 min; **(D)** schematic representation of the self-healing behavior of the hydrogel: two pieces of CMC-OHA hydrogels in different colors (blue: stained with methylene blue; yellow: loaded with curcumin); **(E)** Adhesion, pulling, and recovery of the hydrogel on the porcine skin section; **(F)** finger bending 0°–90° with the loaded hydrogel; **(G)** the hydrogel was injected into the uterus and adapted to the uterine shape through the vagina of ICR mice with a 1-mm-diameter catheter.

One of the main advantages of self-healing hydrogels is their inherent injectability. It helps reduce discomfort for both patients and clinicians when applying biomaterials. As shown in Figure 3A, a 1-mL syringe with an inner diameter of 1.5 mm was used to aspirate a pre-gel solution mixed with Rejuveno dye, and after gelation, the hydrogel was extruded by pressing the syringe and deposited onto paper, demonstrating its injectability. In a practical situation to prevent IUA, the hydrogel would be injected through the cervical canal, which provides limited space for injection. Here, the ability to pass through the narrow catheter ensures that their application does not cause pain or discomfort to the patient. Once they were injected into the mold, they adapted to the structure of the mold, showing their potential to cover every corner of the uterus after injection (Figure 3B). After gelation, they were crushed and reinserted into the mold. It was observed that the CMC-OHA hydrogels self-healed into a star shape after 1 h at room temperature under wet conditions, indicating their self-healing properties. In addition, two differently stained gels were cut into two pieces and re-cross-linked together. After 1 h of incubation in saline, the interface was clearly blurred, and the new glue blocks obtained by complete fusion of the cut surfaces after 6 h further demonstrated their good self-healing properties (Figure 3D). The hydrogel appears faded in color after the self-healing process, as shown in Figure 3D, due to the changes in light scattering and curcumin release after incubation in saline. These properties are crucial for hydrogels intended to be injected through narrow catheters. Even if their structure is destroyed during injection, they self-heal and adapt to the uterine cavity, covering it completely once inside the uterus.

Female ICR mice were further injected *in vivo* to demonstrate injectability. Cur@CMC-OHA hydrogel was injected through the mouse vagina. A catheter (1.5 mm outer diameter; 1 mm inner diameter) was used for injection. As shown in Figure 3G, Cur@CMC-OHA hydrogel was successfully injected into the mouse uterus. After removing from the uterus, it matched the shape of the uterine cavity. This experiment showed that Cur@CMC-OHA hydrogel could be easily injected and adapted to the shape of the cavity.

Cur@CMC-OHA hydrogel has good adhesive power. When Cur@CMC-OHA hydrogel was adhered to the pig skin section, it adhered well to the two sections of pig skin, pulled and recovered, and stretched with the movement of the pig skin without falling off or breaking (Figure 3E). When Cur@CMC-OHA hydrogel was applied to the index finger joint and the index finger was flexed freely to 90°, the hydrogel did not fall off or break (Figure 3F). These two results showed that the hydrogel could adhere well to the skin due to the amino and phospholipid groups on the skin surface. When the hydrogel was in contact with the skin, some of the aldehydes in the hydrogel would react with the amino groups on the skin in a Schiff-base reaction; the hydrogel had a large number of hydroxyl groups, which would also form hydrogen bonds with the amino groups on the skin. In addition, the hydrogel itself contained a large number of unreacted amino groups, which would react with the phospholipids on their skin surface to a certain extent.

### 3.3 Cur@CMC-OHA hydrogel induces endometrial regeneration in a mouse endometrial acute injury model

**FIGURE 4:**
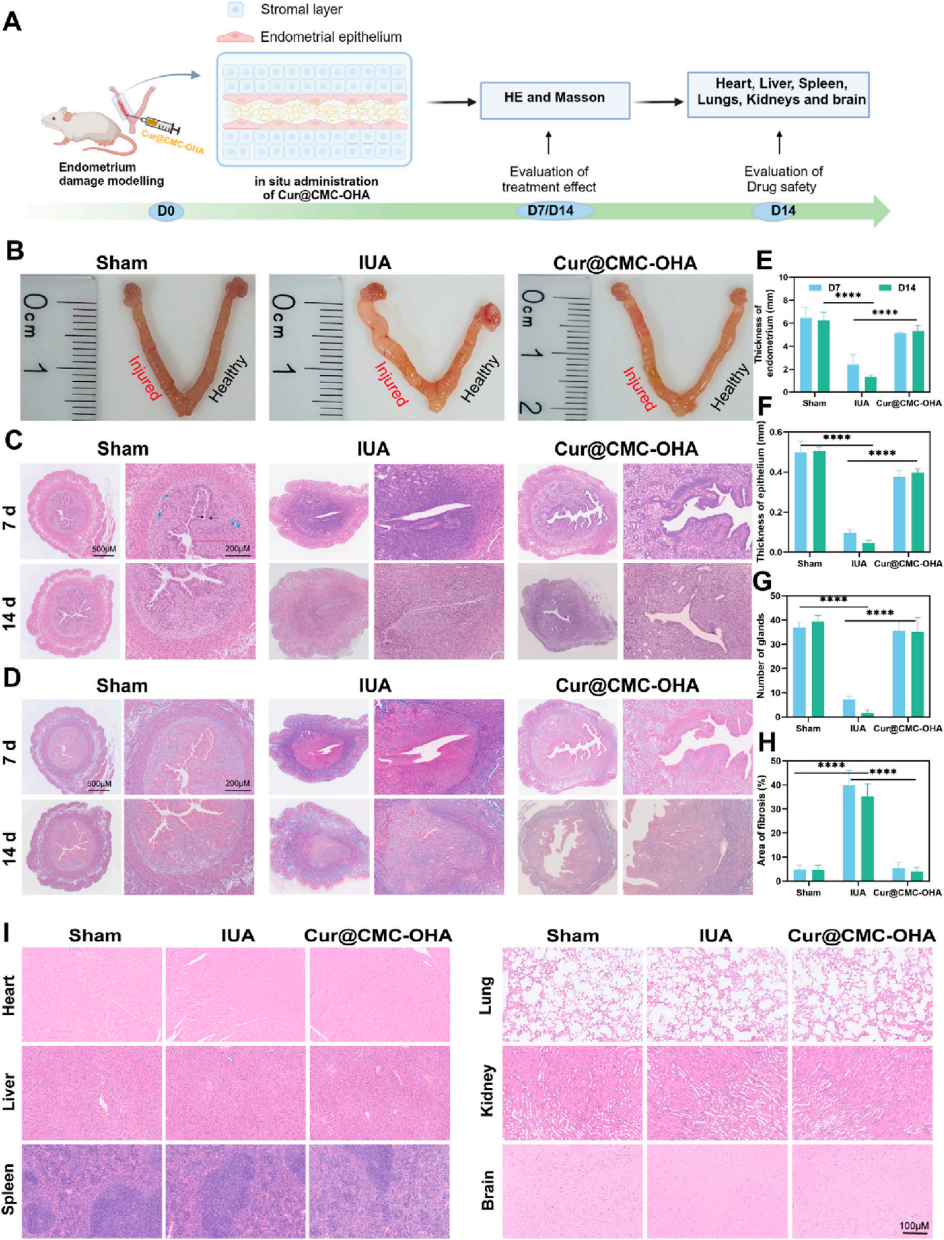
Endometrial regeneration induced by Cur@CMC-OHA hydrogel in a mouse model of uterine adhesions and proved to be safe. **(A)** Schematic diagram of *in vivo* animal experimental procedures; **(B)** representative images of the uterus on day 14 of treatment; **(C)** hematoxylin and eosin (HE) staining on treatment days 7 and 14 (×5 and ×40; blue arrows represent glands, and black arrows represent the epithelium); **(D)** Masson trichrome staining on treatment days 7 and 14 (×5 and ×40); **(E)** results of endometrial thickness calculated using HE staining on days 7 and 14 (n = 3); **(F)** results of endometrial epithelial thickness calculated using HE staining on days 7 and 14 (n = 3); **(G)** Results showing the number of endometrial glands calculated by HE staining on days 7 and 14 (n = 3), **(H)** results showing endometrial fibrous tissue area calculated using Masson staining on days 7 and 14 (n = 3); **(I)** results of HE staining of the heart, liver, spleen, lungs, kidneys, and brains of mice on day 14 (5 and 40×). Data represent at least two independent experiments with similar results; statistical analysis of one experiment is shown. Mean ± s.d.; *P < 0.05, **P < 0.01, ***P < 0.001, and ****P < 0.0001, one-way ANOVA followed by Tukey’s multiple comparison test.

The mouse IUA model was used as a translational tool in endometrial studies because fibrosis following severe endometrial injury in the IUA model was similar to the histopathological changes observed in severe IUA in humans. The IUA model proved to have a thinner than normal endometrium with fewer glands and increased collagen deposition. As shown in Figure 4B, the IUA uterine injury was markedly narrowed, thinned, and hardened, with decreased elasticity and distal edema. A thinner endometrium, with fewer endometrial glands, almost completely covered by low columnar epithelial cells, was observed on days 7 and 14 after modeling, as shown in Figures 4C,D. In severe cases, the uterine cavity could be completely closed. The thickness and morphology of the endometrium were examined and measured under a light microscope. The number of glands was counted in each of five randomly selected fields of view under high magnification, and the mean value was calculated.

Cur@CMC-OHA gel was injected into the uterine cavity to assess the recovery rate. As shown in Figure 4A, Cur@CMC-OHA gel was injected into the uterine cavity directly after establishing the model of IUA, and the therapeutic effect was evaluated. Treatment of the post-injury uterine horn with Cur@CMC-OHA gel resulted in a relatively normal appearance of the uterine horn after 14 days of treatment (Figure 4B), and a significant increase in endometrial thickness and the number of glands was observed on days 7 and 14 (Figures 4C,E–G), *P* < 0.001. Endometrial fibrosis was significantly lower in the Cur@CMC-OHA-treated group at days 7 and 14 than in the IUA model group (Figures 4D,G), *P* < 0.001. The extent of endometrial fibrosis was determined using a quantitative image analysis system (Image-Pro Plus software; Media Cybernetics, Bethesda, MD). As shown in Figure 4I, the *in vivo* experiments indicated that Cur@CMC-OHA gel had no significant toxic effects on vital organs (heart, liver, spleen, lungs, kidneys, and brain). Cytotoxicity assays on IK cells were performed in *in vitro* experiments with no toxic side effects, as shown in Supplementary Figure S1. These results convincingly demonstrate that administration of Cur@CMC-OHA gel improves endometrial production on IUA.

### 3.4 Anti-endometrial fibrosis effect and fertility outcomes of Cur@CMC-OHA hydrogel

**FIGURE 5:**
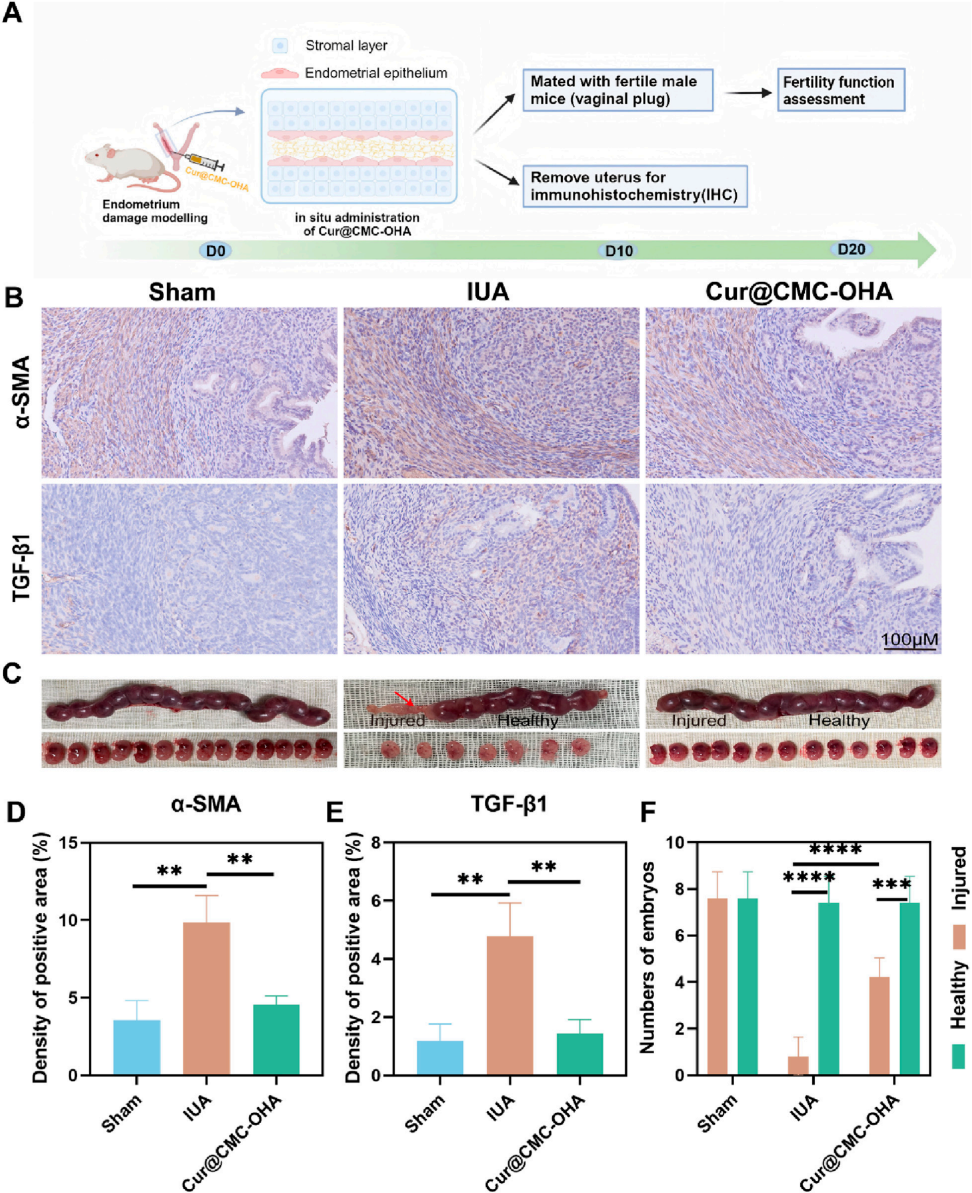
Anti-fibrosis and restoration of reproductive function of Cur@CMC-OHA hydrogels in IUA mice: **(A)** schematic diagram of animal experimental procedures; **(B)** immunohistochemical results of TFG-β and α-SMA in the uterus of mice in each group; **(C)** representative images of embryo implantation; **(D)** analysis of TFG-β positivity in each group, **(E)** analysis of TFG-β positivity in each group, and **(F)** analysis of the number of embryos in each group. Data are expressed as SEM ± mean; n = 3; *P < 0.05, **P < 0.01, ***P < 0.001, and ****P < 0.0001.

TGF-β, expressed mainly in the nucleus and cytoplasm of the hypothalamus of epithelial and stromal cells, is a key regulator of fibrosis formation and promotes the fibrotic process by inducing epithelial–mesenchymal transition (EMT) and the transformation of the cells into myofibroblasts and the deposition of ECM, which promotes the formation of IUA (Hu et al., 2018). These fibroblasts highly express α-SMA and waveform protein. To assess the functional improvement after Cur@CMC-OHA hydrogel treatment in IUA mice, the experimental procedure is shown in Figure 5A. Female mice with damaged uterine horns were injected with the Cur@CMC-OHA hydrogel, and the expressions of TGF-β and α-SMA in the IUA group were significantly higher than those in the surgical group, and the results are shown in Figures 5B,D,E.

We found that there was no embryo implantation in damaged uteri in the IUA group, but embryos were found to implant into the uterus and develop into the late stage of gestation in both the control group and the Cur@CMC-OHA hydrogel group. In addition, the number of embryos was significantly reduced in the IUA group, whereas treatment with Cur@CMC-OHA hydrogel significantly increased the number of embryos, as shown in Figures 5C,F. These results suggest that *in situ* injection of Cur@CMC-OHA hydrogel is effective in decreasing the fibrosis of uterine tissues and restoring the reproductive function of the damaged endometrium.

### 3.5 *In vivo*, Cur@CMC-OHA hydrogel inhibits fibroblast differentiation and EMT for IUA treatment via the TGF-β1/smad3 pathway

**FIGURE 6:**
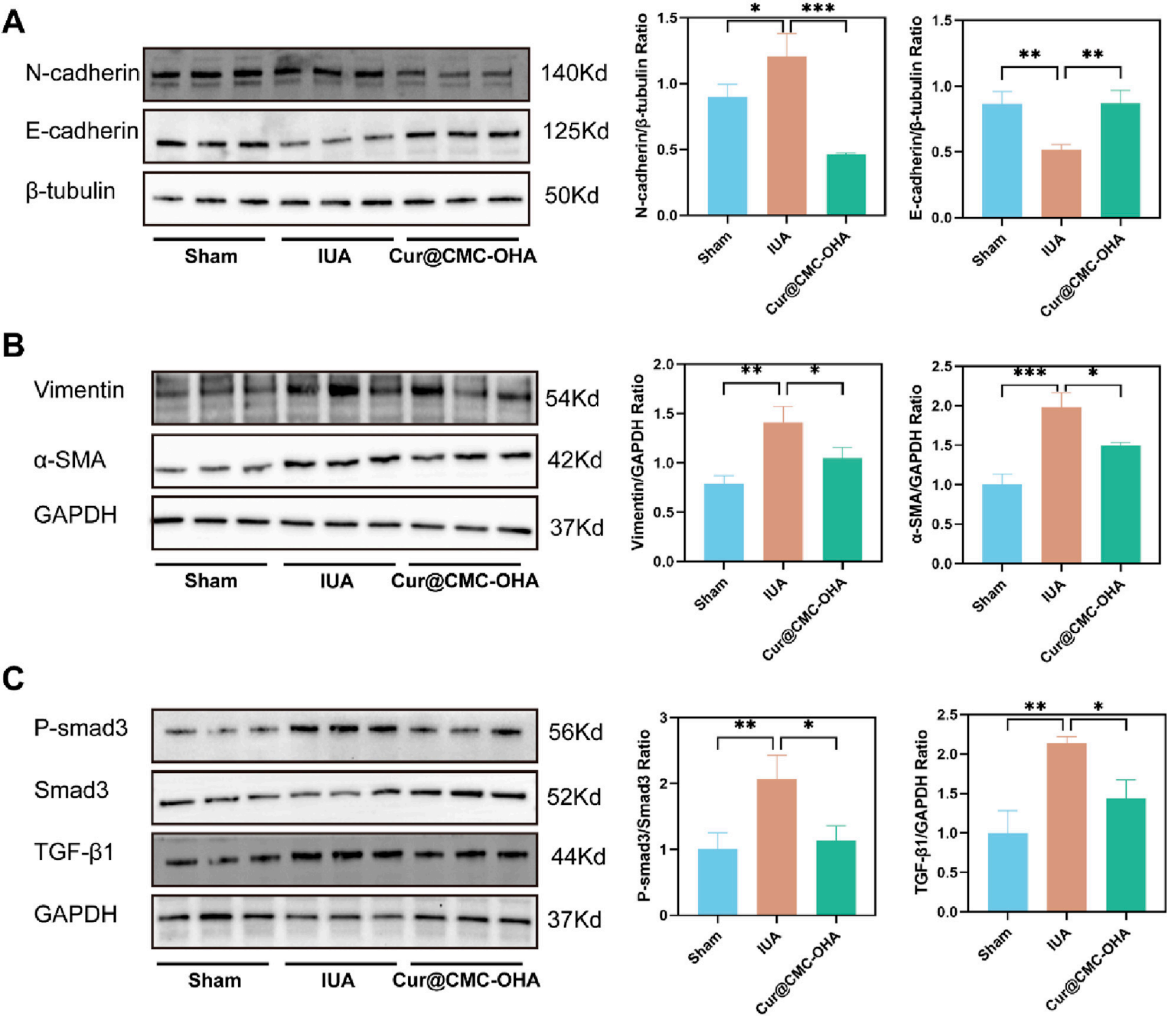
Effect of Cur@CMC-OHA hydrogel in IUA mice. **(A–C)** N -cadherin, E-cadherin, vimentin, α-SMA, p-Smad3, Smad3, and TGF-β1 protein levels were determined (n = 3). Western blot results were semi-quantified by densitometry. Data are presented as means ± standard deviation in the scatter plots. *P < 0.05, **P < 0.01, ***P < 0.001, and ****P < 0.0001.

Fibroblast differentiation and epithelial–mesenchymal transition have been well documented to be associated with uterine adhesions. α-SMA is a marker for myofibroblasts, which secrete a large amount of the extracellular matrix, including collagen. In addition, the expression of vimentin increases during fibroblast differentiation (Weiskirchen et al., 2019). To explore the mechanism of action of Cur@CMC-OHA hydrogel in IUA, we examined the expressions of α-SMA and vimentin. Compared with the model group of mice, the protein expression of all the above indicators was significantly reduced after Cur@CMC-OHA hydrogel administration (Figure 6B). This suggests that Cur@CMC-OHA hydrogel can ameliorate uterine fibrosis in mice by inhibiting fibroblast differentiation. EMT is a reversible process in which epithelial markers (E-cadherin) are lost or downregulated, and mesenchymal markers (N-cadherin) are gained, resulting in the loss of attachment polarity of basement membranes and reduced adhesion at intercellular junctions (Xu et al., 2020). Cur@CMC-OHA hydrogel reversed the decreased expression of E-cadherin and elevated expression of N-cadherin after uterine injury (Figure 6A). This also reflects that Cur@CMC-OHA hydrogel can reverse EMT to achieve antifibrotic effects. In IUA injury-treated mouse uteri, Cur@CMC-OHA hydrogel effectively inhibited TGF-β1 production, suppressed p-smad3, and elevated Smad3 in uterine tissues (Figure 6C). It is widely accepted that TGF-β1 is a key fibrotic factor associated with various fibrotic diseases (Boutanquoi et al., 2020; Lecomte et al., 2023; Meng et al., 2016). TGF-β1 may play a key role in fibrosis of uterine tissues, which is one of the key mechanisms of uterine adhesions. This suggests that the anti-fibrotic effect of Cur@CMC-OHA hydrogel via *in situ* injection may be achieved by inhibiting the TGF-β1/Smad3 pathway.

## 4 Conclusion

In conclusion, injectable, biodegradable and self-healing Cur@CMC-OHA hydrogel is designed to prevent postoperative IUA. Hyaluronic acid is a widely distributed component of the extracellular matrix of human tissues with good biocompatibility and biodegradability. Oxidized hyaluronic acid (OHA) is a product of hyaluronic acid oxidation, and the active aldehyde group in its molecule can self-assemble with the amino group in CMC in a Schiff base reaction to form a hydrogel. The composition ratio of the hydrogel is closely related to the excellent properties of the hydrogel, so one of the important tasks of this study is to find out the appropriate ratio of the hydrogel combination. We found that this self-assembled intrauterine hydrogel exhibits good injectability, self-healing, biodegradability, biocompatibility, along with some compressive strength and adhesion, and gradually breaks the Schiff base bond to release the drug slowly. Cur@CMC-OHA hydrogel improved endometrial production, leading to an increased number of glands, thicker endometrium, and lesser area of endometrial fibrosis compared to those in the IUA group. The expressions of TGF-β1 and α-SMA in immunohistochemical staining of the endometrium were also found to be upregulated in IUA mice. TGF-β is an important factor in endometrial fibrosis, so we speculate that curcumin may prevent endometrial fibrosis by inhibiting the expressions of TGF-β1 and EMT. All these results suggest that our Cur@CMC-OHA hydrogel effectively inhibited fibrosis of uterine tissues and prevented postoperative IUA. However, this study is only an animal experiment conducted to support our research results, and the anti-fibrotic effect and mechanism of curcumin need to be revealed using cell experiments. In this study, the reproductive function was also performed to assess the recovery of the damaged endometrium, and a significant increase in the number of embryos was found after injection of Cur@CMC-OHA hydrogel, inferring that there was significant recovery. We believe that this injectable, self-healing, and stress-resistant hydrogel will be a new candidate for effective prevention of postoperative IUA.

## Data Availability

The raw data supporting the conclusions of this article will be made available by the authors, without undue reservation.
