# Supplementary material for: Biodegradable and injectable curcumin-loaded hydrogel for the prevention of postoperative intrauterine adhesion
Source: Front Bioeng Biotechnol. 2025 Nov 17;13:1640440. doi: 10.3389/fbioe.2025.1640440 (PMC12665675; doi:10.3389/fbioe.2025.1640440)
Supplement: Supplementary file 1 [file Supplementaryfile1.pdf]

Supporting Information

Biodegradable and injectable curcumin-loaded hydrogel for the prevention of postoperative intrauterine adhesion

Qingmei Yang 1, Han Xu 1, Yang Chen 1, Xiaofen Jin 2*, Qing Wu 1*

1. Department of Gynecology, Yibin First People's Hospital, Yibin, China, 644000.

2. Zhejiang Key Laboratory of Precision Diagnosis and Therapy for Major Gynecological Diseases, Women's Hospital, Zhejiang University School of Medicine, Hangzhou, Zhejiang, China, 310001

*Corresponding author

Corresponding author: Qing Wu, Email: okwq31@163.com; Xiaofen Jin, Email: jxiaofen@zju.edu.cn

Table S1. Gel time of different hydrogel formulations.

CMC/OHA（wt%）	Gel Time (s)	Injectability	Self-healing	Adhesiveness
1/1	/			
1/2	/			
1/3	/			
1/4	/			
2/2	/			
2/3	/			
2/4	/			
3/3	/			
3/4	/			
4/4	/			
5/1	/			
5/2	/			
5/3	/			
5/4	/			
5/8	800	✓	✓	
10/1	/			
10/2	/			
10/3	950	✓		
10/4	350	✓	✓	✓
10/8	75	✓	✓	✓
12/8	40	✓	✓	✓
15/1	/			
15/2	/			
15/3	750	✓		✓
15/4	240	✓		✓
15/8	19	✓	✓	✓

Table S1. After mixing different concentrations of CMC and OHA, C10O4, with a gel formation time of 350s was used for the subsequent experiments due to the difference in the length of the gelation time, taking into account adhesion propertythe and self-healing behaviour of the hydrogel.

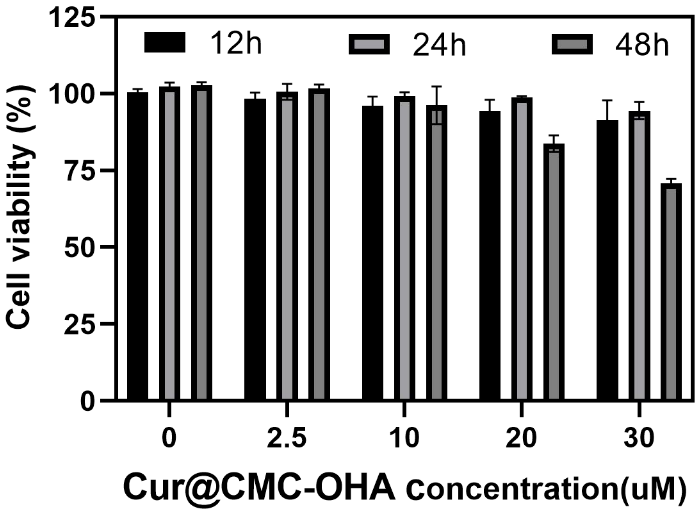

Fig. S1 Assessment of hydrogel viability using the CCK8 assay for IK cells. Cytotoxicity assays on IK cells were performed in in vitro experiments with no toxic side effects of Cur@CMC-OHA gel below 20uM.
